# Family history of diabetes in first-degree relatives and risk of hand surgical diagnoses: a Swedish population-based cohort study from the Malmö Diet and Cancer Study

**DOI:** 10.1136/bmjopen-2026-117616

**Published:** 2026-06-19

**Authors:** Lovisa Lesand, Mattias Rydberg, Peter Nilsson, Lars B Dahlin, Malin Zimmerman

**Affiliations:** 1 Department of Translational Medicine - Hand Surgery, Lund University, Malmö, Sweden; 2 Department of Hand Surgery, Skåne University Hospital, Malmö, Sweden; 3 Department of Medicine, Lund University, Clinical Sciences Malmö, Malmö, Sweden; 4 Department of Orthopedics, Helsingborg Hospital, Helsingborg, Sweden

**Keywords:** Epidemiology, Genetics, Risk Factors

## Abstract

**Abstract:**

**Objectives:**

Diabetes mellitus (DM) has a strong genetic component and is a known risk factor for several hand conditions. We aimed to examine whether DM in first-degree relatives is associated with an increased risk of hand surgical diagnoses.

**Design:**

A retrospective cohort study.

**Setting:**

Data were collected from a population-based cohort in southern Sweden (Malmö Diet and Cancer Study (MDCS)). Family relationships were obtained through the Swedish Multi-Generation Register and healthcare diagnoses from the National Patient Register.

**Participants:**

30 446 individuals (60% female) aged 45–73 years were included from the MDCS.

**Outcome measures:**

The primary outcome was any diagnosis within the ‘diabetic hand’ category; carpal tunnel syndrome, ulnar nerve entrapment, trigger finger, Dupuytren’s disease and thumb base osteoarthritis, identified through International Classification of Diseases, tenth revision codes. Logistic regression models were used.

**Results:**

In the index study population, 4 150 (14%) had ≥1 hand surgical diagnosis. In unadjusted models, DM in the index individual and first-degree relatives was associated with an increased risk of developing hand surgical diagnoses (OR 1.14, 95% CI 1.06 to 1.22). After adjusting for confounders, only DM in the index individual remained significantly associated with the diabetic hand (OR 1.23, 95% CI 1.13 to 1.33). In diagnosis-specific analyses, an association persisted between having ≥1 sibling with DM and Dupuytren’s disease (OR 1.39, 95% CI 1.09 to 1.76), and between having ≥1 child with DM and carpal tunnel syndrome (OR 1.18, 95% CI 1.00 to 1.39).

**Conclusion:**

While DM in the index individual was consistently associated with a higher risk of multiple hand surgical diagnoses, DM in first-degree relatives did not significantly influence the risk after adjustments. Exceptions included an association between ≥1 sibling with DM and Dupuytren’s disease, and ≥1 child with DM and carpal tunnel syndrome. Future research should further explore potential genetic factors contributing to these conditions.

**STRENGTHS AND LIMITATIONS OF THIS STUDY**

- Use of a large population-based cohort with long-term follow-up and high completeness through linkage to national registers.
- Objective ascertainment of family history using the Multi-Generation Register, avoiding recall bias.
- Adjustment for multiple relevant confounders including lifestyle and metabolic factors.
- Inability to distinguish between type 1 and 2 diabetes and lack of data on the number of affected relatives.
- Registry-based outcomes capture diagnoses from specialised care only; potentially missing cases managed exclusively in primary care.

## Introduction

 Peripheral nerve entrapment disorders are common, with carpal tunnel syndrome (CTS) affecting approximately 5% of the population.^1^ CTS and ulnar nerve entrapment, most frequently at the elbow (UNE), represent the most common types of peripheral nerve entrapment disorders in the upper limb and are often classified as idiopathic.^2 3^ Various biological and lifestyle factors may contribute to the development of hand conditions, including diabetes mellitus (DM), smoking, alcohol consumption, body mass index (BMI) and occupational hazards.^4^,^6^ UNE has been associated with male sex, smoking and mechanical stress,^7 8^ whereas CTS is more frequently observed in women, older adults and individuals with obesity.^4 9^ Excessive alcohol intake and DM are risk markers for Dupuytren’s disease (DD).^6^

DM is a key risk factor for several hand surgical disorders, including DD, CTS, UNE, trigger finger (TF) and possibly thumb base osteoarthritis (CMC-1 OA). As a result, multiple studies suggest that these diagnoses collectively fall under the term ‘the diabetic hand’.^10^,^12^ As a rapidly growing global health issue, DM affected 14% of adults aged ≥18 years in 2022, which is an increase from 7% in 1990. Consequently, the number of individuals with DM-related complications continues to rise.^11 13^

Beyond metabolic and environmental influences, genetic predisposition may play a crucial role in developing these conditions and could directly or indirectly impact the pathogenesis. It is known that family history is a risk marker for diseases such as DM, cancer, cardiovascular disease and autoimmune disorders.^14^,^18^ Family history reflects the interplay between shared genetics, the physical and social environment, as well as lifestyle factors. Research on twins and families has demonstrated a strong hereditary component in CTS,^19^ and genome-wide association studies (GWAS) have identified genetic loci linked to CTS, TF and DD.^20^,^23^ Notably, DD has been estimated to have 80% heritability^24^ and may be triggered by a trauma, further highlighting the complex interaction between genetic predisposition and external factors in disease development.^25^ As previously mentioned, DM, which has a strong genetic background, significantly increases the risk for all the conditions mentioned.^11 26 27^ However, whether DM in first-degree relatives influences the risk of developing hand surgical diagnoses remains unexplored.

### Aim

To investigate whether objective family history of DM in first-degree relatives (parents, siblings and children) increases the risk of developing diagnoses related to the diabetic hand.

## Method

### Study population

The Malmö Diet and Cancer Study (MDCS) is a population-based cohort from southern Sweden, including 30 446 individuals aged 45–73 years enrolled between 1991 and 1996, as described in previous publications.^28^,^32^ Data on age, sex, BMI, DM, alcohol intake and smoking were collected within the MDCS through self-administered questionnaires. We based our assessment of participants’ alcohol consumption on the guidelines provided by the National Board of Health and Welfare in 2023. Excessive use of alcohol was defined as consuming 10 or more standard drinks per week, or four or more standard drinks per occasion at least once a month. These thresholds are the same for both men and women. One standard drink was calculated as equivalent to 12 g of pure alcohol.^33^ The MDCS provides data on smoking habits among participants. Individuals who currently smoked at the time of data collection were included in the smoking group.

Additionally, information on diagnoses within the diabetic hand category and DM was obtained through linkage to the Swedish National Patient Register (NPR), established in 1962 and with national coverage since 1987, that has been validated in multiple studies. The NPR covers healthcare provided in hospitals and specialised care, and it has a high coverage rate due to the mandatory reporting requirement for healthcare providers.^34 35^ Both prevalent DM at baseline and incident DM during the follow-up period were included in the variable representing diabetes status, and both type 1 and 2 diabetes were classified within the same group (DM). The diagnoses of common diabetic hand problems were selected based on International Classification of Diseases, tenth revision (ICD-10) codes^36^; CTS (G56.0), UNE (G56.2), TF (M65.3), DD (M72.0) and CMC-1 OA (M18.X).

The Swedish Multi-Generation Register (MGR) was used to identify familial relationships between index individuals and their first-degree relatives (parents, siblings and children).^37^ The register includes approximately 11 million individuals registered as residents in Sweden and documents biological relationships across generations. Index persons are defined as individuals born in 1932 onwards and being alive in 1961. Information on mothers is available for 97% and on fathers for 95% of these individuals, ensuring comprehensive coverage for identifying first-degree relatives.^37^ Cross-linkage with MDCS and NPR was performed using the unique Swedish personal identification numbers. The MGR has contributed data to various prior studies.^38 39^ For each category (parent, sibling, child), we created a binary variable indicating ≥1 first-degree relative with DM; counts of relatives within a category (eg, multiple siblings) were not captured due to lack of data. All participants provided informed consent for inclusion in the MDCS. The authors had access to a pseudonymised, linked dataset and full access to all variables included in the analyses.

### Statistical methods

Using data from the MGR and MDCS, we investigated the relationship between the family burden of DM diagnoses and the risk of developing hand surgical diagnoses through logistic regression analysis in the index individual. Descriptive data were presented with categorical and nominal variables reported as numbers (%) and continuous data as medians (IQR; 25th–75th percentiles). The distribution of continuous variables was assessed visually using histograms and by examining summary statistics (mean and median). The results from the logistic regression were presented with p values, ORs and 95% CIs. A p value of <0.05 was considered statistically significant. All analyses were conducted using IBM SPSS Statistics for Windows, V.28.0 (IBM Corp, Armonk, New York).

In the first model, no adjustments for confounding factors were made. In the second model, we adjusted for known risk factors, including age, sex, BMI, DM, smoking and alcohol consumption. In the adjusted analyses, 1888 individuals were excluded due to missing data on one or more covariates (age, sex, BMI, smoking or alcohol consumption).

### Patient and public involvement

None.

## Results

A total of 30 446 index individuals from MDCS baseline were included in the study. The median age was 59 years (IQR 52–65), and 18 326 (60%) of the participants were female (table 1). The median BMI was 25 (IQR 23–28) kg/m^2^, and 6821 (22%) of the participants were active smokers. Regarding alcohol consumption, the median intake was 7 g/day (IQR 2–15), and 5802 (19%) of individuals reported excessive alcohol use. Further, table 1 continues to describe the study population, where 6745 (22%) had DM, 3133 (10%) had ≥1 parent with DM, 3215 (11%) had ≥1 sibling with DM and 3748 (12%) had ≥1 child with DM. In total, 8482 (28%) of individuals had ≥1 first-degree relative with DM. The registry provided data regarding whether there was a relative with the diagnosis in respective categories or not but did not include information on how many relatives there were. A diagnosis included in the diabetic hand category was observed in 4159 (14%) of the study population. CTS was the most common condition, affecting 1436 (5%) of individuals, followed by CMC-1 OA, 1620 (5%), and TF, 1129 (4%). DD was observed in 663 (2%) participants, and UNE was present in 371 (1%) participants.

**Table 1:** Descriptive characteristics of the study population, including the prevalence of DM and family history of DM, and diabetic hand conditions.

	All (n=30 446)
Sex (female; n, %)	18 326 (60)
Age (years)	59 (52–65)
BMI (kg/m^2^)	25 (23–28)
Current smokers (n, %)	6821 (22)
Alcohol (g/day)	7 (2-15)
Excessive use of alcohol (n, %)	5802 (19)
DM index (n, %)	6745 (22)
DM≥1 parent (n, %)	3133 (10)
DM≥1 sibling (n, %)	3215 (11)
DM≥1 child (n, %)	3748 (12)
DM≥1 parent, sibling and/or child (n, %)	8482 (28)
Diabetic hand index (n, %)	4150 (14)
CTS (n, %)	1436 (5)
UNE (n, %)	371 (1)
TF (n, %)	1129 (4)
DD (n, %)	663 (2)
CMC-1 OA (n, %)	1620 (5)

Continuous variables are presented as median (IQR), and categorical variables as number (percentage).

BMI, body mass index; CMC-1 OA, thumb base osteoarthritis; CTS, carpal tunnel syndrome; DD, Dupuytren’s disease; DM, diabetes mellitus; TF, trigger finger; UNE, ulnar nerve entrapment.

Table 2 presents the prevalence of diagnoses related to the diabetic hand among individuals with DM in different familial subcategories. Among individuals with DM, 16% of those classified as index cases had a diagnosis consistent with the diabetic hand. In comparison, 15% of those with a parent, sibling and/or child with DM were affected.

**Table 2:** Prevalence of the diabetic hand among index individuals and their first-degree relatives (parent, sibling and/or child) with DM

Type of familial group with DM	Diagnosis related to the diabetic hand (n %)		Total with DM (n)
	Yes	No	
Index	1091 (16)	5654 (84)	6745
Parent	478 (15)	2655 (85)	3133
Sibling	474 (15)	2741 (85)	3215
Child	557 (15)	3191 (85)	3748
Parent, sibling and/or child	1250 (15)	7232 (85)	8482

Presented as number and percentage. Percentages refer to the proportion within each familial group with DM.

Familial DM variables indicate ≥1 affected relative within category.

DM, diabetes mellitus.

The unadjusted analysis revealed strong associations between DM in first-degree relatives and the risk of developing diabetic hand conditions (table 3). Having ≥1 parent (p=0.005), child (p=0.019) or any first-degree relative (p<0.001) with DM was associated with an increased risk. A near-significant association was also observed for having ≥1 sibling with DM (p=0.052). Furthermore, DM in the index individual was strongly associated with developing diabetic hand conditions in the same individual (p<0.001).

**Table 3:** Unadjusted and adjusted associations between DM in first-degree relatives and diagnoses within the diabetic hand

Type of familial group with DM	Unadjusted		Adjusted	
	P value	OR (95% CI)	P value	OR (95% CI)
Parent	**0.005**	1.16 (1.05 to 1.29)	0.357	1.05 (0.94 to 1.18)
Sibling	0.052	1.11 (1.00 to 1.23)	0.530	1.04 (0.93 to 1.16)
Child	**0.019**	1.12 (1.02 to 1.24)	0.101	1.09 (0.98 to 1.21)
Parent, sibling and/or child	**<0.001**	1.14 (1.06 to 1.22)	0.099	1.07 (0.99 to 1.15)
Index individual	**<0.001**	1.30 (1.21 to 1.40)	**<0.001**	1.23 (1.13 to 1.33)

The table presents OR with 95% CI for the presence of any diabetic hand diagnosis in relation to DM in first-degree relatives and in the index individual. The results are presented both in unadjusted models and in models adjusted for age, sex, BMI, smoking and excessive alcohol use. In the adjusted analyses, 1888 individuals were excluded due to missing data on one or more covariates. P values <0.05 indicate statistical significance and are marked as bold.

Familial DM exposures are binary indicators for ≥1 affected relative in the specified category.

BMI, body mass index; DM, diabetes mellitus.

After adjusting for age, sex, BMI, smoking and excessive alcohol use, the association between having DM and an increased risk of developing a diabetic hand disorder remained significant (p<0.001). In contrast, no significant associations were observed between diabetic hand diagnoses and having ≥1 parent (p=0.357), sibling (p=0.530) or child (p=0.101) with DM (table 3).

When analysing the diagnoses separately, unadjusted significant associations were observed for certain familial relationships. Having ≥1 parent with DM was significantly associated with an increased risk of CTS (p=0.006) and TF (p=0.006). Having ≥1 sibling with DM was linked to a higher risk of DD (p=0.011) and UNE (p=0.012). Additionally, DM in a child was significantly associated with an increased risk of CTS (p=0.016). When considering DM in ≥1 first-degree relative (parent, sibling or child), a significant association was found for CTS (p<0.001). In both unadjusted and adjusted analyses, DM in the index individual was consistently associated with a higher risk of several hand surgical diagnoses, except for the diagnosis CMC-1 OA, where no such association was observed in the adjusted data (table 4).

**Table 4:** Associations between DM in index individual and first-degree relatives, with specific hand diagnoses

	Unadjusted		Adjusted	
	OR (95% CI)	P value	OR (95% CI)	P value
Index with DM				
DD	1.24 (1.04 to 1.48)	**0.018**	1.24 (1.03 to 1.5)	**0.026**
CTS	1.43 (1.27 to 1.61)	**<0.001**	1.32 (1.16 to 1.5)	**<0.001**
TF	1.46 (1.27 to 1.66)	**<0.001**	1.46 (1.27 to 1.69)	**<0.001**
UNE	1.55 (1.24 to 1.94)	**<0.001**	1.35 (1.05 to 1.73)	**0.017**
CMC-1 OA	1.16 (1.03 to 1.3)	**0.012**	1.05 (0.92 to 1.19)	0.488
≥1 parent with DM				
DD	1.17 (0.92 to 1.48)	0.207	1.11 (0.86 to 1.44)	0.411
CTS	1.26 (1.07 to 1.48)	**0.006**	1.02 (0.86 to 1.21)	0.821
TF	1.29 (1.08 to 1.54)	**0.006**	1.02 (0.84 to 1.24)	0.810
UNE	1.21 (0.88 to 1.65)	0.241	0.95 (0.69 to 1.32)	0.773
CMC-1 OA	1.02 (0.87 to 1.21)	0.782	1.11 (0.93 to 1.33)	0.234
≥1 sibling with DM				
DD	1.34 (1.07 to 1.68)	**0.011**	1.39 (1.09 to 1.76)	**0.008**
CTS	1.14 (0.96 to 1.34)	0.127	0.99 (0.83 to 1.18)	0.912
TF	1.05 (0.87 to 1.27)	0.637	0.88 (0.72 to 1.07)	0.199
UNE	1.45 (1.08 to 1.94)	**0.012**	1.19 (0.88 to 1.61)	0.264
CMC-1 OA	0.93 (0.79 to 1.1)	0.403	0.93 (0.78 to 1.12)	0.467
≥1 child with DM				
DD	0.80 (0.62 to 1.03)	0.081	0.95 (0.73 to 1.24)	0.711
CTS	1.20 (1.03 to 1.4)	**0.016**	1.18 (1.0 to 1.39)	**0.045**
TF	1.06 (0.89 to 1.27)	0.517	1.10 (0.91 to 1.33)	0.325
UNE	1.01 (0.74 to 1.38)	0.958	1.04 (0.74 to 1.45)	0.817
CMC-1 OA	1.15 (1.0 to 1.33)	0.056	1.01 (0.87 to 1.18)	0.887
≥1 any first-degree relative with DM				
DD	1.09 (0.92 to 1.29)	0.323	1.17 (0.98 to 1.39)	0.087
CTS	1.24 (1.11 to 1.39)	**<0.001**	1.10 (0.98 to 1.24)	0.115
TF	1.14 (1.0 to 1.29)	0.054	1.01 (0.88 to 1.16)	0.864
UNE	1.17 (0.93 to 1.45)	0.175	1.01 (0.8 to 1.28)	0.928
CMC-1 OA	1.03 (0.92 to 1.15)	0.621	1.00 (0.89 to 1.13)	0.929

OR, 95% CI and p values are presented for unadjusted and adjusted logistic regression models. Adjusted for age, sex, BMI, smoking and excessive alcohol use. In the adjusted analyses, 1888 individuals were excluded due to missing data on one or more covariates**. **P values <0.05 indicate statistical significance and are marked as bold.

BMI, body mass index; CMC-1 OA, thumb base osteoarthritis; CTS, carpal tunnel syndrome; DD, Dupuytren’s disease; DM, diabetes mellitus; TF, trigger finger; UNE, ulnar nerve entrapment.

## Discussion

In this large population-based cohort with national registry linkage study, we investigated whether DM in first-degree relatives was associated with an increased risk of developing hand surgical diagnoses related to the diabetic hand, specifically CTS, UNE, TF, DD and CMC-1 OA.

The results support previous research indicating that individuals with DM have an increased risk of developing diabetic hand conditions, even after adjusting for potential confounders, such as age, sex, BMI, smoking and alcohol consumption.^45 10^,^12^ The strongest associations were observed for CTS, TF, DD and UNE. In contrast, no association was found for CMC-1 OA, suggesting that other risk factors, such as BMI and the metabolic syndrome, may play a more significant role.^40 41^

DM in first-degree relatives significantly increased the risk of developing various diabetic hand conditions in index individuals before adjustments for confounders. This applied to all types of first-degree relatives included in this study: parent, sibling or child, and any of the parent, sibling or child. When adjusting for confounders, most of the significant associations weakened. Only DM in ≥1 sibling or child had a significant association with an increased risk of developing DD (sibling) and CTS (child). The attenuation of these associations after adjustment highlights the role of confounding factors, such as age, BMI and lifestyle behaviours in mediating this relationship.

Family environment and habits are often inherited and have the potential to determine lifestyle behaviours in children and adolescents. Habits, such as diet, exercise, alcohol consumption and smoking, as well as BMI, are greatly influenced by the parents’ behaviours.^42^,^44^ This may suggest that shared genetic predispositions for DM in first-degree relatives do not directly translate into an increased risk for these hand disorders. Obesity is a hereditary trait that often runs in families and is strongly linked to diabetes risk. This suggests that overweight/obesity (BMI) plays an important role in the association between the hand diagnoses studied and diabetes, and that part of this association is at least in part mediated through BMI or mechanisms associated with obesity. The relationship between obesity and diabetes is complex and multifactorial; both conditions share strong genetic components that may interact, and obesity itself is a well-established risk factor for type 2 DM.^45^ It is possible that individuals with obesity have more healthcare contacts, which could contribute to higher detection rates of diabetes. Moreover, elevated BMI has been identified as an independent risk factor for the development of UNE regardless of diabetes status, raising the possibility that BMI might independently contribute to DD. This highlights the complex interplay between hereditary predisposition and metabolic factors and is an interesting area for future research.^46^

Still, genetic contribution is not necessarily excluded. Given the known hereditary component of conditions such as CTS and DD,^19^,^25^ genetic predispositions may still play a role, potentially through indirect mechanisms such as shared metabolic risk factors or structural variations in connective tissue.^47^ Previous studies suggest that the pathogenesis of CTS may involve a deletion of the peripheral myelin protein 22 (*PMP22*) gene on chromosome 17.^47^ At the same time, other findings indicate that components of the extracellular matrix may play a key role in the pathogenesis, with genetic susceptibility loci linked to extracellular matrix composition identified.^22^ The observed association between having ≥1 sibling with diabetes and DD aligns with studies indicating a genetic predisposition to DD.^24^ An increased sibling recurrence risk in DD has previously been explained by a genetic cause.^25^ DM in siblings may reflect shared genetic factors that are less prominent in parent–child relationships.

A limitation is that familial DM exposures were coded as binary indicators for ≥1 affected parent, sibling or child. We lacked data on the number of relatives within each category. Thus, potential clustering patterns based on the number of affected relatives could not be evaluated.

Data on bilateral disease manifestations were not available in this study. Previous studies have shown that bilateral involvement is associated with greater disease severity and may indicate a stronger underlying genetic background.^22 48 49^ Therefore, data on bilateral cases would have enabled further exploration of possible hereditary contributions to disease development. Likewise, surgical treatment reflects a higher disease severity,^3 48 50^ and future studies could investigate whether treatment modality is associated with familial aggregation or genetic susceptibility. Age at disease onset would also have been of interest to investigate, as hereditary forms are often associated with earlier onset.^51^

Taken together, current evidence supports DM as an important risk marker for several hand surgical conditions, while the contribution of familial diabetes appears to be more complex and likely influenced by shared environmental and lifestyle factors. Our findings add to this field by suggesting that family history of diabetes in first-degree relatives does not independently increase risk after accounting for key confounders, except for DD and CTS.

Future research should aim to disentangle genetic and environmental contributions by incorporating genetic data such as genome-wide association studies or polygenic risk scores, alongside detailed lifestyle and metabolic profiling. Longitudinal studies with information on disease onset and severity may further clarify potential hereditary patterns. Such approaches may help guide more targeted prevention strategies and improve understanding of the underlying mechanisms of the diabetic hand.

### Strengths and limitations

A key strength of our study is the use of comprehensive, linked national register data from the MGR, NPR and the local MDCS, allowing for detailed (objective) family and health information, not based on subjective recall. The large sample size enhances the generalisability of our findings, and the adjustment for multiple confounders strengthens the validity of our conclusions. The unique 10-digit Swedish personal identification number assigned to every citizen enables the cross-linking of registers and facilitates large-scale epidemiological studies.^52^

Some limitations of this study should be acknowledged. First, its observational nature limits causal interpretations. Despite adjustments, residual confounding from unmeasured factors such as occupational exposure or detailed glycaemic control may still influence the results.^11^ We were unable to distinguish between type 1 and 2 DM due to the design of the original cohort and limitations in diagnosis coding, which is a limitation given their distinct etiologies and risk profiles. Type 1 DM is more strongly associated with specific genetic factors, whereas type 2 DM involves a more complex interplay between genetic predisposition and environmental or lifestyle factors.^3853^,^55^

Further, both prevalent DM at baseline and incident DM during the follow-up period were included in the variable representing diabetes status. The index DM group therefore represents individuals with a relatively high overall prevalence and incidence of diabetes. Although stricter inclusion criteria could have been applied, the intention of this study was to explore a more general association and to capture potential shared genetic predispositions, making the exact timing of diabetes onset less critical.

In the present study, we did not have access to data on obesity among first-degree relatives, which limits the ability to disentangle shared genetic from environmental or lifestyle-related influences.

Additionally, reliance on registry-based diagnoses may lead to misclassification or missed subclinical cases, and self-reported data on confounders increases the risk of reporting bias. The exclusion of individuals with insufficient Swedish language skills may also introduce selection bias, potentially limiting the generalisability of the findings to more diverse populations. Furthermore, the study’s population-based design, relying on epidemiological data, makes it challenging to distinguish direct genetic effects from shared environmental and lifestyle factors. To better assess genetic contributions, future research could incorporate twin studies, polygenic risk scores or GWAS data. Lastly, until 1997, the NPR only recorded diagnoses from hospital-based inpatient clinics, which may have underestimated the true prevalence of certain conditions, as cases managed solely in primary care were not included.^34^

## Conclusion

DM is a significant risk factor for several hand surgical diagnoses, particularly CTS, TF, UNE and DD. While DM in first-degree relatives initially showed a significant association with multiple hand conditions, this effect diminished after adjustments. However, a potential association remained between having ≥1 sibling with diabetes and DD, as well as between having ≥1 child with diabetes and CTS. Shared lifestyle factors, as well as genetics, may play a role in the development of the diabetic hand.

## Data Availability

Data may be obtained from a third party and are not publicly available.
