# Supplementary material for: Family history of diabetes in first-degree relatives and risk of hand surgical diagnoses: a Swedish population-based cohort study from the Malmö Diet and Cancer Study
Source: BMJ Open. 2026 Jun 19;16(6):e117616. doi: 10.1136/bmjopen-2026-117616 (PMC13289026; doi:10.1136/bmjopen-2026-117616)
Supplement: Supplementary file 1 [file bmjopen-2026-117616.reviewer_comments.pdf]

PEER REVIEW HISTORY

BMJ Open publishes all reviews undertaken for accepted manuscripts. Reviewers are asked to complete a checklist review form and are provided with free text boxes to elaborate on their assessment. These free text comments are reproduced below.

ARTICLE DETAILS

Title (Provisional)

Family History of Diabetes in First-Degree Relatives and Risk of Hand Surgical Diagnoses: A Swedish Population-Based Cohort Study from the Malmö Diet and Cancer Study

Authors

Lesand, Lovisa; Rydberg, Mattias; Nilsson, Peter; Dahlin, Lars; Zimmerman, Malin

---

VERSION 1 - REVIEW

**Reviewer**	**1**
**Name**	**Habibi, Danial**
**Affiliation**	**Isfahan University of Medical Sciences**
**Date**	**15-Mar-2026**
**COI**	**None**

- It is recommended to present the results in the abstract using statistical indicators and numerical values.

- Describe the method used to examine the normality of the data.

- Explain how the sensitivity analysis was performed for the linear and logistic regression models.

**Reviewer**	**2**
**Name**	**Feng, Bing**
**Affiliation**	**Pennington Biomedical Research Center, Louisiana State University System**
**Date**	**01-Apr-2026**
**COI**	**None**

In this manuscript, Lesand et al In this manuscript, Lesand et al examined whether diabetes in first-degree relatives increases hand surgery risk. They found that only diabetes in the

individual, not relatives, was significantly associated after adjustment, except for sibling diabetes with Dupuytren's disease and child diabetes with carpal tunnel syndrome. The study concludes that personal diabetes, rather than family history, largely drives hand surgical risk. The following minor changes would be really appreciated.

1. It would be valuable to perform a sensitivity analysis separating type 1 and type 2 diabetes, especially considering the age distribution, as this may influence the interpretation of hand disease risk.

2. It would be valuable to perform a sensitivity analysis separating type 1 and type 2 diabetes, especially considering the age distribution, as this may influence the interpretation of hand disease risk.

3. While the influence of BMI and lifestyle on familial associations is discussed, the relative contributions of genetic versus environmental and behavioral factors could be clarified more explicitly.

4. It is recommended to add a part in which the authors can summarize all the progress of current research results in this field, and put forward their views on future research in this direction, and point out the guide for follow-up researchers in conclusion.

VERSION 1 - AUTHOR RESPONSE

Reviewer 1

Dr. Danial Habibi, Isfahan University of Medical Sciences
Comments to the Author:

- It is recommended to present the results in the abstract using statistical indicators and numerical values.

  Reply: We have revised the abstract to include key numerical results, including odds ratios and 95% confidence intervals.

- Describe the method used to examine the normality of the data.

  Reply: We have clarified in the Methods section that the distribution of continuous variables was assessed visually using histograms and by comparing summary statistics.

- Explain how the sensitivity analysis was performed for the linear and logistic regression models.

  Reply: We thank the reviewer for this comment. Formal sensitivity analyses were not performed. Instead, we compared unadjusted and adjusted models to assess

the robustness of the associations. This has now been clarified in the Methods section and also acknowledged as a limitation.

Reviewer 2

Dr. Bing Feng, Pennington Biomedical Research Center, Louisiana State University System
Comments to the Author:

In this manuscript, Lesand et al In this manuscript, Lesand et al examined whether diabetes in first-degree relatives increases hand surgery risk. They found that only diabetes in the individual, not relatives, was significantly associated after adjustment, except for sibling diabetes with Dupuytren's disease and child diabetes with carpal tunnel syndrome. The study concludes that personal diabetes, rather than family history, largely drives hand surgical risk. The following minor changes would be really appreciated.

Reply: Thank you for your valuable and constructive comments. We address them individually in detail below.

1. It would be valuable to perform a sensitivity analysis separating type 1 and type 2 diabetes, especially considering the age distribution, as this may influence the interpretation of hand disease risk.

   Reply: We appreciate this important suggestion and agree that it would have been a valuable analysis. Unfortunately, we were unable to distinguish between type 1 and type 2 diabetes due to limitations in the available registry data and diagnostic coding. This limitation has now been explicitly clarified in the Methods and Discussion sections.

2. While the influence of BMI and lifestyle on familial associations is discussed, the relative contributions of genetic versus environmental and behavioral factors could be clarified more explicitly.

   Reply: We have now expanded the Discussion to more clearly describe the interplay between genetic predisposition with environmental and lifestyle factors, including BMI and shared family behaviours.

3. It is recommended to add a part in which the authors can summarize all the progress of current research results in this field, and put forward their views on future research in this direction, and point out the guide for follow-up researchers in conclusion.

   Reply: We have expanded the Discussion and Conclusion sections to provide a clearer summary of current knowledge and to highlight future research directions.

VERSION 2 - REVIEW

Reviewer **2**

Name **Feng, Bing**

Affiliation **Pennington Biomedical Research Center, Louisiana State University System**

Date **13-May-2026**

COI

The submission has been greatly improved according to the reviewers' comments, and it is worth publishing.
